# A Comprehensive Review of Xanthan Gum-Based Oral Drug Delivery Systems

**DOI:** 10.3390/ijms251810143

**Published:** 2024-09-21

**Authors:** Buddhadev Layek

**Affiliations:** Department of Pharmaceutical Sciences, School of Pharmacy, College of Health and Human Sciences, North Dakota State University, Fargo, ND 58105, USA; buddhadev.layek@ndsu.edu; Tel.: +1-701-231-7906; Fax: +1-701-231-8333

**Keywords:** controlled release, drug delivery, matrix tablets, polysaccharide, xanthan gum

## Abstract

Xanthan gum (XG) is an exopolysaccharide synthesized by the aerobic fermentation of simple sugars using *Xanthomonas* bacteria. It comprises a cellulosic backbone with a trisaccharide side chain connected to alternative glucose residues in the main backbone through α (1→3) linkage. XG dissolves readily in cold and hot water to produce a viscous solution that behaves like a pseudoplastic fluid. It shows excellent resistance to enzymatic degradation and great stability throughout a broad temperature, pH, or salt concentration range. Additionally, XG is nontoxic, biocompatible, and biodegradable, making it a suitable carrier for drug delivery. Furthermore, the carboxylic functions of pyruvate and glucuronic acid offer a considerable opportunity for chemical modification to meet the desired criteria for a specific application. Therefore, XG or its derivatives in conjunction with other polymers have frequently been studied as matrices for tablets, nanoparticles, microparticles, and hydrogels. This review primarily focuses on the applications of XG in various oral delivery systems over the past decade, including sustained-release formulations, gastroretentive dosage forms, and colon-targeted drug delivery. Source, production methods, and physicochemical properties relevant to drug delivery applications of XG have also been discussed.

## 1. Introduction

XG is an exopolysaccharide synthesized mainly by the aerobic fermentation of simple sugars using *Xanthomonas* bacteria [1,2]. These bacteria are plant pathogens detected on the leaf surfaces of Brassica vegetables such as cabbage, broccoli, brussels sprouts, cauliflower, kale, and turnip. It was discovered in the early 1950s as a result of a new USDA industrial gum screening program by Allene Rosalind Jeanes and her colleagues at the Northern Regional Research Laboratory (NRRL) in Peoria, Illinois, USA. After dextran (in the early 1940s), it was the second microbial polysaccharide to be marketed. In the early literature, XG was referred to as polysaccharide B-1459. It was first manufactured commercially by the Kelco Company (now CP Kelco) of San Diego, California, USA, under the trademarks Keltrol (food grade) or Kelzan (industrial grade, primarily petroleum industry) [3]. In 1969, the US Food and Drug Administration (Fed. Reg. 345376) approved XG as a food additive, and in 1980, the European Union (E-number 415) did as well [4].

Chemically, XG comprises a β-(1→4) linked D-glucose backbone and a side chain of β-D-mannose-(1→4)-β-D-glucuronic acid-(1→2)-α-D-mannose bonded to the alternating glucose moieties at C-3. XG’s average molecular weight typically varies between 1 × 10^6^ and 2 × 10^7^ Da, based on the biosynthetic and interchain association conditions [4,5]. It is easily dissolved in hot and cold water but remains insoluble in most organic solvents. The XG aqueous solution exhibits high intrinsic viscosity and behaves as a pseudoplastic fluid even at very low concentrations. The viscosity of the XG solution could be modulated by altering polymer concentration, temperature, pH, and salt concentration [6]. Nevertheless, the changes in viscosity are entirely reversible since they occur from molecular conformation variations rather than degradation [7].

XG shows high resistance to pH changes, allowing it to stay stable in acidic and alkaline environments [2]. Also, XG often has superior heat stability than most water-soluble hydrocolloids, and its viscosity recovers after heat treatment stages during food processing, such as pasteurization or sterilization [8]. Thus, their rheological characteristics remain steady regardless of whether the finished products are stored at an ambient temperature, heated, or maintained in a refrigerator. Furthermore, being a natural polymer, XG is nontoxic, biocompatible, and biodegradable. Therefore, XG is extensively utilized in foods, cosmetics, and pharmaceutical products as an emulsifier, stabilizer, and thickener.

XG is frequently employed as a release-retardant matrix alone [9,10,11,12] or in conjunction with other polymers, including chitosan [13], sodium alginate [14], guar gum [15], and konjac glucomannan [16], in developing controlled-release dosage forms. The presence of β-(1→4) linked D-glucose backbone and the unique side chain structure give XG a strong resistance to enzymatic degradation and ensure that it remains indigestible in the upper part of the gastrointestinal tract (GIT). However, it is partially fermented by the bacterial inhabitants of the colon, making it useful for colon-specific drug delivery [17]. Therefore, XG-based hydrogel has also been used as a vehicle for oral protein and peptide delivery [18].

This review provides an updated summary of the recent advances in XG-based oral drug delivery systems. While several publications discuss the broader applications of XG in drug delivery, this review delves deeper into its role in various advanced oral delivery systems, such as sustained-release formulations, gastroretentive dosage forms, and colon-targeted drug delivery. It provides detailed insights that are not readily available in any previously published articles. XG’s source, manufacturing processes, and physicochemical characteristics pertinent to oral drug delivery applications have also been discussed. The challenges and future prospects of XG and its derivatives as versatile and sustainable biomaterials in various biomedical fields have been highlighted.

## 2. Source, Structure, and Properties

XG is synthesized commercially from *X. Campestris* through aerobic fermentation [4,19]. The production process comprises a multi-stage inoculation preparation followed by fermentation, pasteurization, cell removal, gum precipitation, and separation, drying, milling, and packaging. At first, a small number of preserved bacteria is grown in seed culture media to build sufficient inoculum for large bioreactors. The seed culture is then introduced to the fermentation medium (5% of the total volume) and incubated at 28 °C with agitation. The fermentation medium consists of carbohydrate sources (e.g., glucose, sucrose, starch, sugarcane molasses, etc.), nitrogen sources (e.g., ammonium nitrate, urea, peptone or yeast extract), magnesium salt, phosphates ions, and trace elements. A chelating agent such as citric is often incorporated to maintain the stability of the medium in the presence of iron and other trace elements. All fermentation equipment is appropriately cleaned and sterilized before use, and stringent aseptic procedures are followed throughout the fermentation process to prevent culture contamination.

After fermentation, the broth containing XG, bacteria, and other compounds is pasteurized to kill viable microorganisms. The bacterial cells are then isolated by filtration or centrifugation, and XG is precipitated using ethanol or isopropanol. After precipitation, XG is dewatered mechanically, dried, milled, and packaged in containers with low water permeability. The fermentation yield and the xanthan molecular characteristics depend on several factors, including fermentation mode (batch-wise or continuous), bioreactor type, growth medium composition, and culture parameters such as pH, temperature, and oxygen transfer rate [2,20]. For instance, acetyl and pyruvyl contents can be altered by changing bacterial strain and fermentation conditions [7,17]. On the other hand, culture media supplemented with citric acid leads to XG with more pyruvate [2]. Further, XG biosynthesized at lower temperatures, such as 25 and 28 °C, contained larger acetate levels than those produced at 31 and 34 °C [21]. Similarly, lower temperatures (e.g., 25 °C) also yield high molecular weight XG. The physical properties of commercially available XG are listed in Table 1.

**Figure 1:**
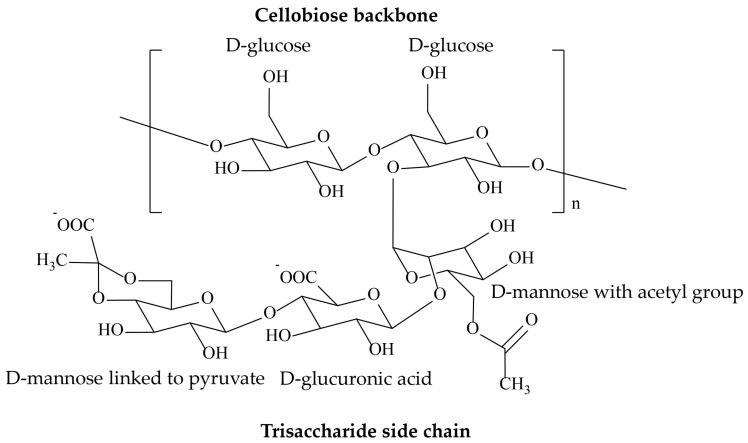
Chemical structure of xanthan gum.

XG is a branched polysaccharide containing repeating pentasaccharide subunits of D-glucose, D-mannose, and D-glucuronic acid in a 2:2:1 molar ratio [22]. The primary structure comprises a β-(1→4) linked D-glucose backbone and a side chain of β-D-mannose-(1→4)-β-D-glucuronic acid-(1→2)-α-D-mannose bonded to the alternating glucose moieties at C-3 (Figure 1) [23]. In the side chains, some of the terminal D-mannose units are connected to the ketone group of pyruvic acid, while the internal D-mannose residue can be acetylated at the C-6 position. Between 30 and 50% of the pyruvate is substituted, while 60–70% of the internal mannose residues are acetylated [17].

**Figure 2:**
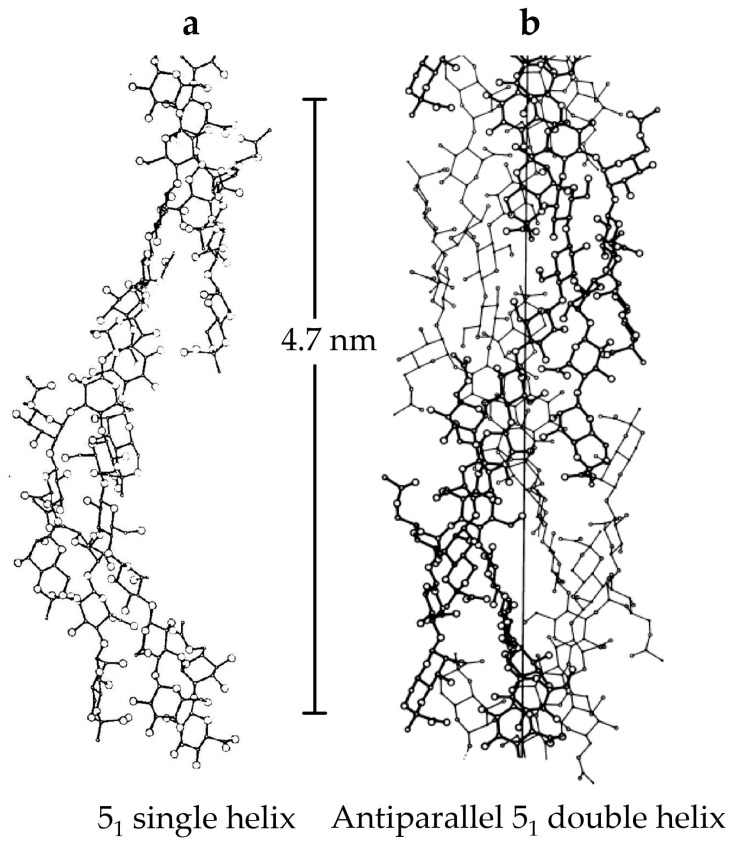
Ordered structures proposed from computer-modelling of X-ray fiber diffraction data for xanthan: (**a**) single helix (**b**) antiparallel double helix. Both structures have 5-fold symmetry and a pitch of 4.7 nm, derived directly from the experimental diffraction patterns. Reprinted with permission from Ref. [3]. Copyright 2019, Elsevier.

The secondary structure of XG molecules in aqueous media has been widely investigated. Depending on the conditions under which the molecule is characterized, it can exist in orderly or disordered states. In the ordered conformation, natural xanthan appears as a 5-fold right-handed helix with a 4.7 nm pitch and 1.9 nm diameter (Figure 2) [3,7,24]. Trisaccharide side chains remain closely associated with the polymer backbone, and non-covalent interactions, particularly inter- and intramolecular hydrogen bonds, preserve the overall structural conformation. The secondary structure of XG undergoes a reversible structural transition from an ordered double helix to a disordered coiled structure due to variations in temperature, ionic strength, and degree of ionization of acetyl and pyruvyl contents [4,25]. At ambient temperatures, the XG chains maintain a helical configuration. However, the hydrogen bonds weaken above the transition temperature, and the chain adopts a more flexible disordered coil structure that increases solution viscosity [26,27,28,29]. The concentration of polymers and the presence of salt both affect the transition temperature. The transition happens at around 40 °C for aqueous solutions containing 0.1–0.3 g/dL [30]. Adding salt to the XG solution causes charge screening, which eliminates electrostatic repulsions between carboxylic groups in the side chains. This stabilizes the helical conformation, which makes it more rigid and shifts the transition temperature to higher values [26,31]. Briefly, high temperatures increase the likelihood of xanthan molecules changing to a disordered coil shape, whereas the number of ions influences the formation of a more structured and stable helix configuration. It has been shown that in an acidic medium, XG molecules adopt a helical configuration [32], while as the pH of the medium is raised, the XG chains transition from a helical to a disordered coil-like structure [33].

## 3. Applications

The extensive use of XG can be ascribed to its unique properties, which include high aqueous solubility, high intrinsic viscosity, viscoelastic behavior, outstanding stability across a broad range of temperatures, pH values, or salt concentrations, resistance to enzymatic destruction, and interaction with other polymers. Along with these advantageous physicochemical characteristics, XG is also nontoxic, biocompatible, and biodegradable, making it an ideal polymer for a range of pharmaceutical and biological applications, including drug delivery and tissue engineering [34,35]. Furthermore, the carboxyl groups of pyruvate and glucuronic acid offer considerable opportunities for chemical modification to satisfy specific application needs [23,36]. The following section highlights applications of XG and its composites/derivatives in various oral delivery formulations, including sustained release, gastroretentive, colon-specific, and protein delivery systems.

### 3.1. Sustained Release System

The use of XG in modified-release dosage forms is made possible by its slow dissolution rate and significant swelling in biological fluids. Both water-soluble and insoluble medicines can be successfully loaded into the XG matrix to develop sustained-release formulations. Further, it offers time-independent release kinetics and delays drug release [10]. Sparingly soluble or insoluble drugs are released owing to matrix erosion, whereas soluble pharmaceuticals are primarily released through diffusion.

As a sole rate-retarding polymer, XG has effectively sustained drug release from matrix systems, especially for slightly water-soluble drugs. In a recent study, Venkatesh et al. [37] developed XG-based sustained-release tablets of nateglinide to treat type-II diabetes. The FTIR spectroscopy analysis confirmed the compatibility of nateglinide and various formulation excipients. XG-containing formulations exhibited sustained drug release profiles dependent on the polymer concentration. Matrix tablets with 10% (*w*/*w*) XG followed the first-order release kinetics and extended nateglinide release for 24 h. Moreover, sustained-release formulations demonstrated a greater extent of drug release and 3.4 times higher bioavailability than immediate-release tablets. XG-based matrices have also been explored for the controlled delivery of theophylline [38], indomethacin [11], diclofenac sodium [39], pentoxifylline [12], venlafaxine hydrochloride [40], isosorbide-5-mononitrate [10], propranolol hydrochloride [41], and quetiapine fumarate [42].

XG has also been chemically modified to enhance its drug delivery potential. For instance, poly (acrylamide) grafted XG-based matrix tablets were prepared for oral delivery of diclofenac sodium [43]. Microwave-assisted or ceric-induced grafting techniques were used to synthesize poly(acrylamide) grafted xanthan derivative. The grafting percentage was higher with the microwave-assisted technique than the ceric-induced method. The grafting percentage increased as microwave power and/or exposure time increased. It was revealed that erosion of the graft polymers changed directly with the percent grafting, while swelling of the polymer varied inversely with the grafting percentage. The matrix tablets formulated with grafted polymers displayed faster drug release than the unmodified XG tablets.

**Figure 3:**
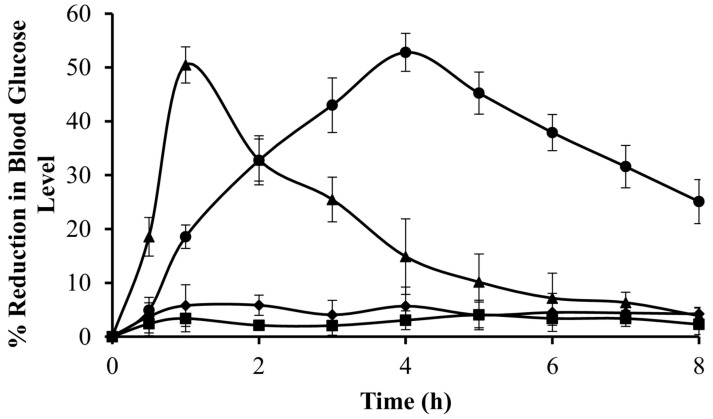
Preclinical efficacy of XGD6 formulation (acetylated hydrogel particles of CEXG: CMXG at 1:2 ratio). Key: diabetic control (♦, received only distilled water), negative control (■, received formulation without drug), pure repaglinide suspension (▲, 4 mg/kg body weight), and XGD6 formulation (●, repaglinide-loaded XGD6 hydrogel particles containing an equivalent amount of repaglinide). Reprinted with permission from Ref. [44]. Copyright 2022, Elsevier.

Despite being a successful biopolymer for drug delivery, XG has little control over drug release due to its low particle hydration properties. The synthesis of ionically cross-linked hydrogel particles is similarly challenging because of XG’s limited gelation capability in the presence of metal ions. Therefore, XG must be chemically modified to address these issues and incorporate additional functionalities to enhance its pharmaceutical applications. In a recent study, Patel, Maiti, and Moorthy [44] synthesized carboxymethyl and carboxyethyl derivatives of XG and evaluated their performance as controlled-release matrices. Repaglinide-loaded hydrogel microparticles were formulated using carboxyethyl XG (CEXG), carboxymethyl XG (CMXG), or their combination. Hydrogel particles manufactured with CEXG alone demonstrated low drug entrapment efficiency (43%) compared to CMXG and CMXG/CEXG hydrogel particles. However, the entrapment efficiency of the CEXG derivative improved upon incorporating CMXG and showed maximum entrapment (92%) at a CEXG/CMXG ratio of 1:2. Particles prepared with CEXG alone swelled more rapidly in a 0.1 M HCl (pH 1.2) medium than in a phosphate buffer (pH 6.8). On the other hand, CEXG/CMXG hydrogel particles swelled slowly in both media; however, the swelling rate was greater in the phosphate buffer. An in vitro drug release investigation in simulated gastrointestinal fluid established that around 97% of the encapsulated drug was released within 4 h. Also, the surface acetylation of the microparticles significantly decreased the swelling rate and prolonged release for 8 h without matrix disintegration. Most importantly, acetylated particles were capable of reducing blood glucose levels in an animal model for 8 h (Figure 3).

Using a single polymer often fails to fulfill all product requirements for oral controlled-release formulations. One of the alternatives for enhancing polymer performance is to use polymer blends with complementary properties. A mixture of XG and konjac glucomannan was employed to make sustained-release matrix tablets [16]. It was demonstrated that the strong intermolecular hydrogen bonds formed by the two polymers in the gel phase effectively inhibit drug diffusion, ensuring sustained drug release. Likewise, the use of XG and hydroxypropyl methylcellulose (HPMC) in combination to create modified-release matrix tablets of metoprolol succinate was studied [45]. Furthermore, the net negative charge of XG permits it to interact with positively charged polymers such as chitosan, providing additional drug release control. Due to its advantages over single polymer matrices, XG and chitosan combinations have been frequently used in controlled-release formulations.

An XG/chitosan-based polyelectrolyte complex was used to encapsulate neomycin sulfate to control drug release and protect against the harmful effects of the free drug [46]. The electrostatic interactions between carboxylic groups of XG and amino groups of chitosan resulted in the formation of the XG/chitosan hydrogel. When neomycin was incorporated into the XG/chitosan hydrogel, additional bond formation occurred between the free amino groups of neomycin and the carboxyl groups of XG. XG/chitosan/neomycin hydrogen bonding and the ionic interactions between polymers were established through FTIR analysis. However, in the basic medium, these bonds break (pKa of neomycin is 9.2). Thus, a large amount of water penetrates among the macromolecular chains, leading to larger swelling of the hydrogel in simulated intestinal fluid (SIF, pH 7.2) than in simulated gastric fluid (SGF, pH 2), indicating that drug release is increased at basic pH. A study in healthy Wister rats supported the XG/chitosan/neomycin formulation’s preventive impact by improving several biochemical measures (e.g., creatinine, C-reactive protein, urea, and uric acid) and histological alterations usually induced by free neomycin administration. Nevertheless, a previous study showed that the XG/neomycin complex was stable in acidic pH, and drug release occurred only at basic pH [47].

To produce PECs, both polymers must have opposite charges and be sufficiently ionized. Different formulation parameters and preparation conditions can impact the extent of ionization, charge density, charge distribution, and the flexibility of polymer chains, thereby influencing the formation and drug delivery potential of PECs. Ćirić and colleagues [48] investigated the combined impact of pH-modifying agents (acetic, hydrochloric, or lactic acid) and initial pH value (3.6, 4.6, or 5.6) on the chitosan/XG PECs formation, solid state characteristics, and in vitro drug release kinetics. The strongest ionic interactions were seen in PECs made with acetic acid at pH 3.6, but the extent and strength of ionic interactions steadily declined with increasing pH in both the aqueous and solid states. The pH and type of pH adjusting agent strongly impacted particle size (100–250 µm) and dry PECs yield. However, PECs formulated using acetic acid at pH 4.6 and 5.6 exhibited more rehydration in a phosphate buffer (pH 7.2) and permitted continuous drug release for up to 10 h. The same research team also examined the influence of drug loading methods (before or after mixing polymers) and chitosan/xanthan mass ratios (i.e., 1:1, 1:2, and 1:3) on polymer cross-linking and drug release profiles [49]. Pre-PEC drug incorporation at pH 4.6 and a 1:1 mass ratio of chitosan to xanthan resulted in significant cross-linking. However, the yield and drug entrapment of PECs made with chitosan/xanthan mass ratios of 1:2 and 1:3 were better.

**Figure 4:**
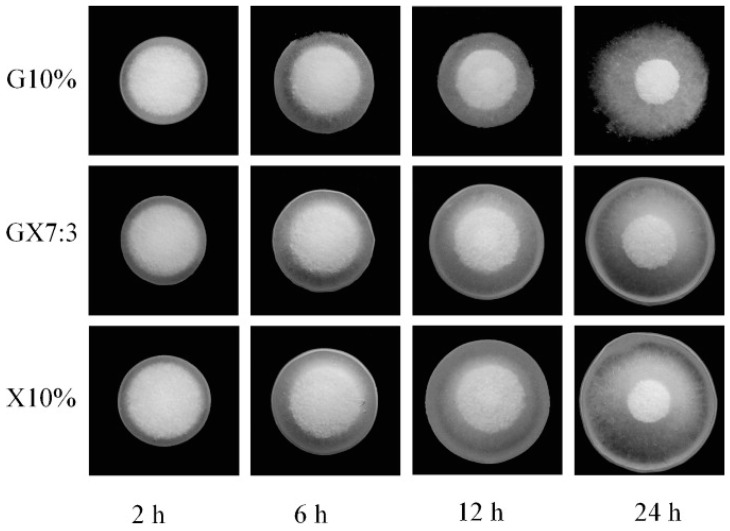
Photographs taken of G10% (galactomannan 10%), GX7:3 (physical mixtures of galactomannan and xanthan gum at 7:3 ratio), and X10% (xanthan gum 10%) systems after swelling for 2, 6, 12 and 24 h in the dissolution test. Reprinted with permission from Ref. [50]. Copyright 2012, Elsevier.

Galactomannan and XG binary mixtures were utilized as a hydrophilic matrix for theophylline’s controlled release [50]. Matrix tablets were formulated with a 10% (*w*/*w*) hydrophilic polymer of either galactomannan, XG, or their physical mixtures at the ratios of 7:3, 5:5, and 3:7. A dissolution study revealed that about 87.7% and 91.4% theophylline was released from the XG (10% *w*/*w*) and galactomannan (10% *w*/*w*) matrix tablets, respectively, after 24 h. Galactomannan/XG matrices, on the other hand, demonstrated longer-lasting drug release than matrix tablets made of either galactomannan or XG alone, demonstrating synergistic interactions between galactomannan and XG in regulating drug release. The most prolonged drug release profile (75.5% at 24 h) was observed in galactomannan/XG matrix tablets at a 7:3 ratio. Except for tablets containing only XG, where the drug release mechanism included polymer relaxation and diffusion, theophylline in vitro release profiles followed anomalous transport or non-Fickian diffusion. Further, the morphological alterations of the swollen tablets revealed that all of the formulations rapidly expanded after being placed in the dissolution medium, with a steady increase in tablet size. Tablets formulated with 10% (*w*/*w*) galactomannan started to lose integrity after 6 h of hydration as a result of the hydrodynamic stress caused by the dissolution device. However, XG (10% *w*/*w*) and galactomannan/XG (7:3 ratio) matrix tablets were capable of maintaining their integrity during the 24 h dissolution test (Figure 4). Due to isotropic swelling, the tablets can keep their original shape, so swelling only alters the matrix’s size without deforming the tablet. Additional examples of XG-based sustained-release oral formulations are given in Table 2.

### 3.2. Gastroretentive Systems

Due to short residence times, drugs with a limited absorption window in the upper part of the GIT frequently have poor bioavailability when administered in a conventional dosage form. The bioavailability of these drugs can be improved by using controlled-release drug delivery systems with a long stomach residence duration. As a result, many gastroretentive formulations have been developed to lengthen the residence time of drugs in the upper GIT. Furthermore, gastroretentive delivery systems are helpful in treating gastric diseases such as ulcers or *Helicobacter pylori* (*H. pylori*) infections. XG alone or combined with other polymers has been extensively used to manufacture various gastroretentive formulations. It does not provide the ability to float but instead traps the formed gas due to its gelling property, thus improving gastroretention [57,58]. Additionally, XG possesses mucoadhesive characteristics that allow its use in gastro-retentive and other drug delivery systems such as nasal, buccal, ophthalmic, and skin formulations [59,60]. XG has also been used to prepare size-increasing gastroretentive matrix tablets along with various in situ gelling polymers [61].

Diltiazem HCl, a calcium channel blocker, is often prescribed to treat angina pectoris and high blood pressure. It is mainly absorbed from the upper portion of the GIT and experiences extensive first-pass metabolism, leading to low oral bioavailability (30–40%). Gastroretentive floating tablets were formulated utilizing varied concentrations of XG to improve the oral bioavailability of diltiazem HCl [57]. Direct compression was used to manufacture floating matrix tablets with PVP K-30 as a binder and sodium bicarbonate as a CO_2_-generating agent. All the formulations were seen to preserve the integrity of the matrix while floating continuously on the dissolution medium with the desired floating lag time. In vitro dissolution was performed for 12 h at 37 °C using USP basket-type equipment. The concentration of XG and sodium bicarbonate significantly affected the diltiazem release rate. Tablets with a higher XG concentration (60% *w*/*w*) exhibited a lower drug release rate than formulations with a lower concentration of XG (40% *w*/*w*) due to the formation of a thicker gel layer.

Migraines have a diurnal cycle, with headaches being more intense in the morning; hence, the need for antimigraine medication is often felt during the early hours. Conventional antimigraine medication formulations cannot be given to patients before their symptoms worsen since they are asleep at the time. Sumatriptan succinate is the most commonly prescribed migraine medicine, although it has some downsides, including poor oral bioavailability, a shorter plasma half-life, and a bitter taste. As a result, compression-coated sumatriptan succinate floating pulsatile tablets have been developed as an effective treatment for migraine chronotherapy [58]. The floating pulsatile delivery concept was used to enhance gastric resident time of the dosage form. The immediate-release tablet core was prepared using 10% *w*/*w* crospovidone as a super disintegrant. The tablet core was compression coated using polyox WSR205 and an XG mixture to produce the desired pulse lag time. Hydrogen bonds were formed between the polyether chains of polyox WSR205 and water. The polymer then hydrates, producing a superficial gel that progressively disappears as the polymer dissolves. XG has a high swelling capacity involving water absorption and a low degree of erosion owing to polymer relaxation. As a result, a clear relationship between swelling and lag time was found. The formulations with the highest swelling indices were found to have a longer lag time. When the coated tablet was immersed in the dissolving media, the hydrophilic polymeric layer expanded and gradually changed in thickness and consistency. The drug is released when the tablet’s outer shell bursts due to pressure from the swelling after it has reached a limit. Due to the varying swelling and erosion properties of XG and polyox WSR205, the drug release patterns of the polymers alone and in combination varied. An optimized formulation containing 72.72% *w*/*w* polyox WSR205 and 27.27% *w*/*w* XG demonstrated 98.69 ± 2% drug release in a pulsatile fashion with a release lag time of 7 h. Furthermore, the X-ray study revealed long gastric retention (6 ± 0.5 h) of the optimized tablets in healthy humans.

*H. pylori* is a bacterium responsible for chronic stomach infection, which affects about two-thirds of the global population. It is the most prevalent cause of gastritis, which can lead to other gastrointestinal problems such as duodenal and peptic ulcers [62]. Levofloxacin has shown promising results against *H. pylori* strains resistant to metronidazole and clarithromycin [63]. However, the complete eradication of *H. pylori* needs long-term maintenance of high antibiotic concentrations in gastric mucosa. A gastroretentive dosage form can ensure high gastric drug concentration for an extended period. With this aim, size-increasing gastroretentive matrix tablets of levofloxacin hemihydrate were formulated using various in situ gelling polymers such as gellan gum, pectin, sodium alginate, or XG [61]. Cross-linking agents such as aluminum chloride (AlCl_3_) and calcium chloride were utilized to slow the dissolution rate. Levofloxacin’s in vitro release has been demonstrated to be dependent on the cross-linking agent as well as the matrix type. However, tablets prepared with XG exhibited the most extended drug release profile and increased diameter with time. The FTIR and DSC analyses of levofloxacin, XG, and their physical mixture confirmed the lack of interactions between the drug and the polymer.

Calcium carbonate is the most affordable and widely recommended calcium supplement worldwide. It should be noted that while calcium carbonate is soluble in the stomach’s acidic condition, it is practically insoluble in water at a neutral pH. Additionally, calcium can only be absorbed from the duodenum via the active transporter protein calbindin, making calcium absorption site-specific and saturable. Due to these challenges, the traditional oral calcium dosage formulations only have a 25–35% bioavailability [64]. Therefore, an in situ gelling raft-forming system (GRFS) of calcium carbonate was developed to increase gastric retention and thereby enhance oral bioavailability [65]. The goal was to create floating GRFS that could float for at least six hours, show the least amount of buoyancy lag time, and guarantee a controlled but nearly full release of the entire drug with minimal burst release. A simple lattice design was employed to investigate the influence of formulation composition (%XG and %HPMC K100) on buoyancy lag times and calcium carbonate release. It was observed that HPMC K100 M has a favorable effect on buoyancy lag time, while XG was discovered to have a negative effect on buoyancy lag time. Formulations with larger amounts of HPMC K100 M were often found to have higher floating lag times. Similarly, HPMC K100 M impacted the burst release favorably, whereas XG had a negative influence. When xanthan gum comes into contact with an aqueous media, it forms a highly viscous and strong gel network. As expected, increasing the amount of XG enhanced the compactness of the gel matrix while decreasing the porosity, resulting in a minimal burst effect and a controlled release of calcium from the raft. The optimized formulation comprising HPMC K 100 M/XG at a ratio of 65.88 to 34.13 exhibited a short buoyancy lag time of 10.90 ± 0.56 s, low burst release of 20.74 ± 1.08%, and 87.25 ± 1.81% calcium carbonate release in 6 h. In vivo radiographic tests on rabbits revealed that the mean stomach retention time for the optimized formulation was 5.64 h, which was significantly longer (*p* < 0.05) compared to the mean stomach retention time for the marketed solution, which was less than one hour.

Fenoverine is a phenothiazine-based antispasmodic drug prescribed to relieve muscle spasms associated with irritable bowel syndrome (IBS) [66]. However, fenoverine’s shorter half-life (5–7 h) necessitates frequent daily doses, especially for chronic illnesses like IBS. Therefore, a gastroretentive floating drug delivery system of fenoverine was designed to increase its gastric residence time and minimize the fluctuation of plasma drug concentration [67]. Fenoverine floating tablets were manufactured by the direct compression method using XG and sodium alginate as a polymeric matrix. Sodium bicarbonate and citric acid were used as gas-forming agents. The polymers increased the viscosity of the formulation in the dissolution media and prolonged drug release for up to 12 h. The formulation containing 45% XG was the best, releasing 99.6% of the fenoverine in 12 h. It also displayed improved swelling ratios, the necessary drug release kinetics, and floating behavior.

Propranolol is a non-selective β-blocker used for the treatment of hypertension, angina, cardiac arrhythmias, myocardial infarction, essential tremors, hyperthyroidism, anxiety, and migraines [68]. It is highly lipophilic and is almost entirely absorbed after oral administration. Nevertheless, propranolol undergoes extensive first-pass metabolism; only approximately 25% of it typically reaches systemic circulation. It has a short elimination half-life of 3–4 h, requiring three to four daily doses. Propranolol displays pH-dependent solubility with a higher solubility at pH 1.2 than at pH 6.8. It also remains stable at an acidic pH while degrading quickly at an alkaline pH.

Therefore, floating tablets of propranolol HCl were developed using 25%, 30%, and 35% of hydroxypropyl cellulose and XG to achieve sustained drug release in the stomach [69]. Tablets were formulated using a direct compression technique at various drug-to-polymer ratios, and sodium bicarbonate was used as a gas-forming agent. The formulations were found to have a floating lag time of less than three minutes. In vitro drug release from the tablets depended on the amount of release retardant in the tablet, which was reduced with the increase in the polymer percentage. Tablets formulated using XG could prolong drug release for 12 h because of its high molecular weight. Tablets containing 30% XG were the optimal formulation and demonstrated 97.5% drug release at the end of 12 h. On the contrary, tablets prepared with hydroxypropyl cellulose alone were entirely dissolved within 6 h due to their poor viscosity. However, when blended with xanthan gum, it was capable of regulating drug release while maintaining matrix integrity for up to 12 h. In light of this, it can be said that by lowering dosage intervals, propranolol floating tablets can improve patient compliance and therapeutic efficacy.

Dexlansoprazole is a proton pump inhibitor used to treat gastroesophageal reflux disease and erosive esophagitis [70]. The poor bioavailability (40–45%) and short plasma half-life (approximately 1–2 h) of dexlansoprazole following oral treatment support the development of a sustained-release formulation [71]. With this aim, gas propellant controlled-release floating tablets of dexlansoprazole were designed by Sontale et al. [72] to prolong gastric residence time and thus enhance its oral bioavailability. The prepared tablets performed well regarding different post-compressive characteristics such as thickness, hardness, friability, weight variation, and content uniformity. Sodium bicarbonate significantly influences buoyancy lag time, whereas chitosan significantly affects overall floating time and in vitro drug release. Carbopol has a considerable influence on in vitro drug release as well. XG and sodium alginate helped maintain the tablets’ integrity and provided adhesive properties. The optimum formulation showed sustained drug release up to 12 h. A glimpse of a few XG-based gastroretentive formulations is summarized in Table 3.

### 3.3. Colon-Specific Delivery Systems

Colon-targeted drug carriers have attracted considerable attention to treat colon-related diseases such as Crohn’s disease, inflammatory bowel disease (IBD), ulcerative colitis, colon cancer, amebiasis, and other colonic diseases locally with minimal systemic side effects [82]. Additionally, colon-targeted carriers are beneficial for the oral delivery of drugs prone to enzymatic or acidic degradations in the stomach. Nevertheless, traditional oral dosage forms are unsuccessful in delivering drug contents to the colon because of their absorption and/or degradation within the upper segment of the GIT.

Several techniques for colon-specific drug delivery have been developed, including pH-responsive systems, time-dependent release systems, and microbially triggered systems [83]. pH-responsive systems often display unpredictable site specificity of drug release due to high inter- and intrasubject variability and nearly identical pH values of small intestinal and colonic fluids. XG remains undigested in the human stomach or small intestine; instead, it breaks down in the presence of colon enzymes [84]. Hence, XG matrices are utilized for the colonic delivery of drugs while protecting them from the stomach and small intestine environments. Upon entering the colon, the anaerobic microflora breaks them down into smaller monosaccharides, which these microbes can then utilize as a source of energy. These microbes produce a wide variety of reductive and hydrolytic enzymes, which include β-glucuronidase, β-galactosidase, α-arabinosidase, β-xylosidase, azoreductase, deaminase, urea hydroxylase, etc. [85,86].

5-Fluorouracil (5-FU) is an integral part of combination chemotherapy in colon cancer’s palliative and adjuvant treatment. It is usually delivered parenterally due to its variable and incomplete absorption following oral administration. Like other chemotherapeutic agents, 5-FU has a small therapeutic window and can cause severe dose-limiting toxicities, including neutropenia, leukopenia, diarrhea, and mucositis. Therefore, colon-targeted delivery would not only lessen the drug’s systemic toxicity but also demonstrate the intended effect at a lower dose. The compression coating of tablets is an efficient strategy for colonic drug delivery. With this aim, a mixture of XG and GG in different ratios was compression-coated onto rapidly disintegrating 5-FU core tablets [87]. The dissolution studies were carried out in SGF (pH 1.2) for the first 2 h and then in SIF (pH 6.8) with and without rat cecal contents (2 or 4% *w*/*v*) for the remaining time. After 24 h of dissolution, tablets coated with 175 mg of a XG/GG mixture at 20:20, 20:10, and 10:20 ratios released 18 ± 1.23%, 20 ± 1.54%, and 30 ± 1.77% of 5-FU, respectively. It was found that reducing the coat weight to 150 mg did not influence the initial drug release at SGF, though the total amount of 5-FU released after 24 h increased to 25 ± 1.22%, 36.6 ± 1.89%, and 42.6 ± 2.22%, respectively, for tablets coated with a XG/GG mixture at 20:20, 20:10, and 10:20 ratios. However, the presence of rat cecal contents significantly boosted drug release. After 19 h of incubation, drug release from tablets coated with a 10:20 XG/GG gum combination was 67.2 ± 5.23% and 80.34 ± 3.89% in the presence of 2% *w*/*v* and 4% *w*/*v* rat cecal contents, respectively.

Sinha et al. [88] also used Boswellia gum and a XG mixture at different ratios (1:2, 2:1, 1:3, 1:7, and 3:4) to coat 5-FU core tablets for colon-targeted delivery. The dissolution studies were conducted in SGF for the first 2 h and then in SIF (pH 6.8) with 2% *w*/*v* rat cecal contents for the remaining time. The optimum drug release profile (7.47 ± 1.56% in the first five hours) was achieved with a 1:3 Boswellia gum to XG ratio with a minimum coating weight of 230 mg. When the coat weights were increased further to 250 mg, 275 mg, and 300 mg, the resulting drug release was 5.63 ± 0.53%, 5.09 ± 1.56%, and 4.57 ± 0.88%, respectively, in the first 5 h and 96.90 ± 0.66%, 85.05 ± 1.01%, and 80.22 ± 0.35%, respectively, within 24 h. However, a total drug release was accomplished when 2% *w*/*v* of rat cecal contents was present.

Time-dependent colonic delivery systems are based on the idea of deferring drug release until the system moves from the mouth to the colon. The limitation of time-dependent release systems is that they cannot detect changes in upper GIT transit time. The significant fluctuation in stomach retention time in a time-dependent system makes it appear impossible to accurately estimate the location of drug release [89], even though the small intestine transit time is generally consistent and less variable (3  ±  1 h) [90]. Microbially triggered systems generally consist of an immediate-release tablet that is compression-coated with natural polysaccharides digested by the colon’s anaerobic microorganisms. However, several factors can substantially alter the makeup of the human gut ecology, thereby affecting drug release. Furthermore, because of the hydrophilic nature of the polysaccharides, a greater coat thickness is needed to prevent premature drug release. Conversely, though a thicker coating can reduce pre-colonic release, in the absence of specific enzymes or cecal components, they lead to sustained release instead of burst release of the drug after a suitable lag period. Hence, creating a compression-coated tablet that erodes gradually enough to prevent or at least minimize pre-colonic release and then delivers an instantaneous burst release of drug in the colon, regardless of the polysaccharide’s enzymatic metabolism by colonic microflora, is a more sensible approach.

**Figure 5:**
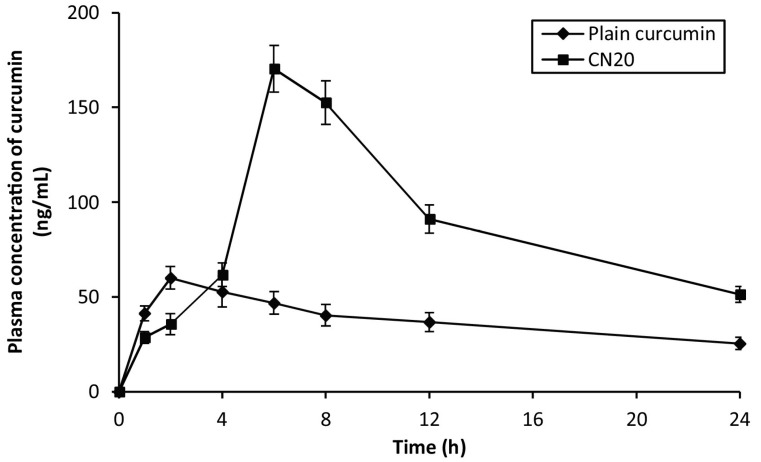
Plasma concentration vs. time curves of pure curcumin and curcumin-loaded PAAm-g-XG nanoparticles (CN20 NPs). Reprinted with permission from Ref. [91]. Copyright 2016, Elsevier.

Curcumin, a naturally occurring polyphenolic compound, is known to have a protective role in IBD patients due to its potent anti-inflammatory and antioxidant effects. However, the beneficial effect of curcumin is often mitigated because of its unfavorable pharmacokinetic profile and low oral bioavailability. Therefore, pH-responsive nanoparticles made of polyacrylamide-grafted-XG (PAAm-g-XG) were developed for colon-specific curcumin delivery [91]. Curcumin-loaded PAAm-g-XG nanoparticles (CN20) with an average diameter of 425 nm were manufactured using the solvent evaporation cross-linking technique. CN20 nanoparticles showed low curcumin release in pH 1.2 and 4.5 buffers, and only about ~8% of the curcumin was released after 3 h (0–2nd hours in pH 1.2 HCl solution plus 2nd–3rd hour in pH 4.5 solution). Curcumin release from CN20 nanoparticles was noticeably faster when the medium’s pH was raised from pH 4.5 to 7.2 (3rd–6th hours). This increased curcumin release could be attributed to the higher solubility of the grafted polymer in alkaline pH. Nevertheless, by the end of the sixth hour, the curcumin release was only approximately 35%. Excellent curcumin release was detected when the pH of the medium was changed to 6.8 (6th–9th hours). Almost 65% of the curcumin was released in the pH 6.8 solution within three hours. When CN20 nanoparticles were incubated at pH 7.4, pores formed due to the ionization of the -COOH groups and the electrostatic repulsion of the ionized groups, which helped release the encapsulated drug. At pH 1.2, no surface holes were seen since the -COOH groups remained unionized. The higher drug release rate during the 6th–9th hours in pH 6.8 solution could be attributed to the development of pores on nanoparticles during the 3rd–6th hours at pH 7.2 and subsequent drug release through these pores. However, maximum curcumin release was achieved in a pH 6.8 medium containing 1% (*w*/*v*) rat cecal content, suggesting microflora-mediated drug release properties of the nanoparticles. In a rat model of IBD, the curcumin nanoparticles were able to lower myeloperoxidase and nitrite levels, prevent weight loss, and diminish colonic inflammation. Additionally, as compared to free curcumin, nanoparticle-encapsulated curcumin had better systemic absorption, as indicated by a roughly 3-fold rise in Cmax and a roughly 2.5-fold improvement in AUC (Figure 5).

Mesalamine is often used to treat ulcerative colitis, especially in patients intolerant to sulfasalazine [92]. But prolonged mesalamine usage can cause many undesirable side effects, including diarrhea, nausea, cramping, flatulence, myalgia, headache, interstitial nephritis, alopecia, and even sometimes myocarditis, pancreatitis, hepatitis, and leukopenia [93,94,95]. These adverse effects are primarily due to the systemic absorption of the drug. Consequently, several colon-targeted formulations have been developed to avoid its absorption in the upper part of the GIT. However, the efficacy of such formulations is compromised because the trigger for drug release is exclusively dependent on gut microflora, which is often disturbed by mesalamine. An innovative strategy was adopted to overcome this limitation by combining mesalamine-containing colon-targeted microspheres with probiotics in a single formulation in which the polymer also functions as prebiotics [96]. Mesalamine microspheres were formulated by emulsion polymerization using GG/XG mixtures. Microspheres were mixed with probiotics that contained *Lacticaseibacillus rhamnosus*, *Lacticaseibacillus acidophilus*, *Saccharomyces boulardi*, and *Bifidobacterium longum*. The results of the dissolution study revealed that 92.56% of the drug from the synthesized microspheres was released into the colon, while a high percentage of drug (over 70%) was released into the SIF for both delayed release and extended release marketed tablets. Most importantly, a comparative analysis of the fecal material, weight gain patterns, and histological examinations of ulcerative colitis rat model confirmed the therapeutic advantages of the co-administration of synbiotics and mesalamine.

It may be possible to obtain a controlled drug release profile by combining time-dependent and pH-sensitive technologies. For example, pH-responsive polymers Eudragit (L100 or S100) were combined with XG to develop colon-targeted indomethacin matrix tablets [97]. Tablets comprising XG alone swelled rapidly in the presence of the release medium, and a substantial portion of the drug was released during the early phase. However, the addition of Eudragit to XG limited swelling of the matrix and resulted in a negligible indomethacin release in the initial stages, followed by a controlled release lasting 14–16 h.

Table 4 lists additional examples of XG-based colon-specific drug delivery systems.

### 3.4. Protein Delivery Systems

The rapid advancement of recombinant DNA technologies allows the synthesis of a wide range of proteins and peptides with varying biological properties. These bioactive proteins and peptides quickly emerge as essential components of various therapies for treating fatal diseases like cancer, diabetes, and heart conditions [107,108,109]. However, despite intensive research, oral administration of these biomolecules remains a major concern for pharmaceutical researchers. Large molecular weight, strong hydrophilicity, degradation by proteolytic enzymes, and first-pass hepatic metabolism all contribute to the limited oral bioavailability of protein therapeutics [110]. It is well known that polysaccharide-based hydrogels are an excellent option for designing protein delivery systems to prevent denaturation during their encapsulation. For instance, bovine serum albumin (BSA) microparticles were formulated using CMXG in an entirely aqueous medium [111]. The synthesized microparticles were discrete, spherical, and had a high entrapment efficiency (82%). However, the higher swelling of the microparticles in the acidic medium contributed to the faster BSA release in the acidic release medium compared to the alkaline medium. The pH of the gum solution used to formulate the microparticles also substantially impacted swelling and subsequent protein release.

**Figure 6:**
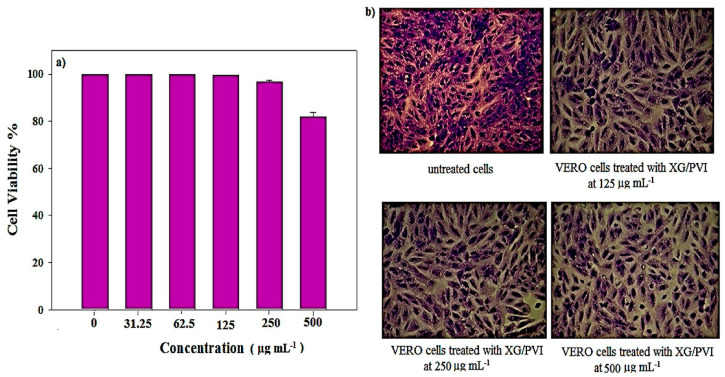
(**a**) The percentage of cell viability of different concentrations of XG/PVI hydrogel sample after 24 h incubation with VERO cell lines and (**b**) microscope examination of VERO cells that were incubated 24 h with different concentrations of hydrogel samples and compared with control cells (untreated cells). Reprinted with permission from Ref. [112]. Copyright 2019, Elsevier.

Recently, an XG/poly (N-vinyl imidazole) (PVI) hydrogel was used for the oral delivery of BSA [112]. BSA loading capacity and encapsulation efficiency of the hydrogels increased as the gelation time and higher BSA concentration but decreased with the increased polymer concentration. The maximum values of BSA loading and encapsulation efficiency were 59.50% and 99.17%, respectively, when an equal weight ratio of XG and PVI were used along with a gelation time of 24 h and a BSA concentration of 75% (*w*/*w*) of total polymer weight. The equal weight ratio of XG to PVI increased the rigidity of the gel matrix, resulting in more loaded BSA on XG/PVI hydrogels. Several formulation factors, including the XG/PVI weight ratios, BSA concentration, and gelation time, influenced the in vitro release of BSA in PBS (pH 7.4). It has been revealed that the maximum BSA release occurred at 120 h when the overall XG concentration, or XG/PVI ratio, increased. This is because an increase in XG concentration may lead to a more entangled system, which would increase the distance between the XG and PVI chains due to the amorphous nature of XG and thereby reduce matrix-free volume. An increase in matrix-free volume enhanced BSA release from the matrix. However, compared to other ratios, the matrix with an equal XG to PVI weight ratio showed less BSA release because the gel matrix’s stiffness increased and its free volume shrank. The synthesized hydrogels were also tested for biocompatibility. Cytotoxicity tests on VERO cells demonstrated that the hydrogels are nontoxic and biocompatible (Figure 6). An SDS-PAGE analysis verified that neither the encapsulation nor release conditions affected the structural integrity of BSA. Therefore, XG/PVI hydrogels showed great potential for oral protein delivery without affecting their structural integrity.

In another study, Abu Elella et al. [18] synthesized biodegradable, a pH-sensitive cross-linked XG-g-PVI/N, N′-Methylene bisacrylamide (MBA) hydrogel for oral BSA delivery. The BSA encapsulation efficiency increased with the increasing percentage of graft yields, BSA, and graft concentration and decreased with the increasing MBA concentration. The swelling and BSA release studies were conducted at pH 1.2 and 7.4, representing stomach and intestinal pH, respectively. At pH 7.4, both swelling rate and cumulative BSA release percentages were quicker than at pH 1.2. Conversely, the cumulative BSA release in pH 7.4 medium increased with the increasing percentage of graft yields and decreased with BSA, MBA, and graft concentrations. The BSA release mechanisms followed the non-Fickian model in both media. The structural integrity of the released BSA was confirmed by SDS-PAGE. The cytotoxicity results on human normal lung cells (Wi38) indicated the good biocompatibility of the cross-linked hydrogel.

A lack of dietary selenium (Se) often leads to many health issues, including Kashin-Beck disease and Keshan disease [113]. Se-containing peptides have emerged as potential dietary Se supplements in the pharmaceutical and food sciences. To this end, XG/lysozyme nanoparticles were developed for the oral delivery of two Se-containing peptides—TSeMMM (STP) and SeMDPGQQ (SHP) [114]. Both STP and SHP nanoparticles displayed negative zeta potentials (−47 mV and −49 mV) and relatively small particle sizes (145 nm and 148 nm, respectively). The encapsulation efficiency of SHP and STP nanoparticles was 34.35% and 41.35%, respectively. Nanoparticles were capable of controlling Se-containing peptides over a prolonged period under SGF. Furthermore, nanoencapsulation significantly improved the stability and antioxidant activity of Se-containing peptides. The apparent permeability coefficient of STP (2.19 × 10^6^ cm/s) and SHP (2.21 × 10^6^ cm/s) significantly increased as a result of the low toxicity of nanoparticles and their enhanced uptake by Caco-2 cells via clathrin-mediated endocytosis. As a result, XG/lysozyme-based nanoparticles are regarded as potential delivery vehicles for Se-containing peptides with applications in the food and pharmaceutical industries.

A recent study evaluated four distinct anionic pH-responsive polysaccharides (XG, hyaluronic acid, propylene glycol alginate, and alginic acid) for their ability to enhance chemical stability and facilitate the sustained release of antimicrobial peptides vancomycin and daptomycin [115]. All of these polysaccharides comprise carboxylic groups that are ionized to generate anionic polyelectrolytes when the pH is close to or above their pKa (XG: glucuronic acid 3.1, pyruvic acid 2.9; hyaluronic acid: glucuronic acid 3.0; propylene glycol alginate and alginic acid: mannuronic acid 3.4, glucuronic acid 3.7). The electrostatic interactions between the anionic carboxylate groups of these polysaccharides and the cationic amine groups of the drugs allow the formation of stable polyelectrolyte complexes. Vancomycin displayed first-order degradation kinetics with a reaction rate constant (kobs) of 5.5 × 10^−2^ day^−1^ when incubated in a pH 7.4 buffer at 37 °C. However, the kobs of vancomycin reduced to (2.1–2.3) × 10^−2^ day^−1^ in the presence of XA, hyaluronic acid, or a propylene glycol alginate-based hydrogel, while kobs remained mostly unaffected in an alginate hydrogel and a dextran solution. Similarly, XG and propylene glycol alginate effectively reduced daptomycin kobs (5.6 × 10^−2^ day^−1^); however, alginic acid had no impact, and hyaluronic exacerbated the degradation rate. These findings show XG and propylene glycol alginate’s protective role in delaying the breakdown of both peptides and hyaluronic acid for vancomycin.

## 4. Conclusions and Future Prospects

XG is one of the most extensively used exopolysaccharides produced by aerobic fermentation of simple sugars using Xanthomonas bacteria. The yield and product characteristics depend on several factors, including bacterial strain, mode of operation, type of bioreactors, culture medium composition, and culture conditions like pH, temperature, and oxygen transfer rate. Therefore, thorough physicochemical and structural characterization is critical to producing XG with desired properties. XG has been extensively used as a pharmaceutical excipient due to its excellent physicochemical characteristics, stability across a broad range of pHs, temperatures, and salt concentrations, biocompatibility, non-toxicity, and low-cost abundance. Most importantly, the US FDA has granted XG Generally Recognized as Safe (GRAS) status for use as a food ingredient.

However, a few disadvantages of XG include inadequate mechanical and thermal properties, variable viscosity, microbial contamination, low shear resistance, and unpredictable degree of hydration. Furthermore, XG slowly disintegrates, especially at high concentrations and in cold water. Therefore, XG is often chemically modified to enhance its physicochemical characteristics such as solubility, swelling and mucoadhesion profile, mechanical and thermal stability, and gelling capacity. Numerous reactive carboxyl and hydroxyl groups make XG amenable to various chemical modifications. The most common chemical modifications are acetylation and pyruvation, grafting and covalent cross-linking, esterification, etherification, oxidation, and peptide conjugation.

In oral dosage forms, XG or its derivatives are frequently utilized as matrix-forming agents for controlled-release, gastroretentive, colon-targeted formulations as well as protein and peptide delivery, alone or in combination with other polymers. XG has also been incorporated recently into several nanoformulations to increase oral bioavailability. Although XG is mainly used for oral delivery, it has also been employed in transdermal, ocular, nasal, parenteral, and rectal formulations. Apart from small molecules and protein and peptide-based therapeutic delivery, XG-based formulations have also been investigated as a vehicle for genetic materials. In a study, XG-functionalized sorbitan monooleate (Span) nanoparticles were formulated for targeted gene delivery to endothelial cells [116]. The synthesized nanoparticles demonstrated long-term stability and efficient protection of the encapsulated DNA. Nanoparticles showed low cytotoxicity and effective DNA transfection ability in vitro and in vivo. The main goal of this study was to target liver sinusoidal endothelial cells (LSECs) by incorporating XG into span nanoparticles. The mannose residues in XG were thought to aid in targeting mannose receptors, which are known to be overexpressed in LSECs. As a scavenger receptor, the mannose receptor recognizes and facilitates the absorption of a wide range of glycoconjugate ligands. However, it was found that XG span nanoparticles are internalized by LSECs and vascular endothelial cells lacking mannose receptors following systemic administration. These nanoparticles could be useful for targeting the vascular endothelium of the liver, lung, and kidney, avoiding reticuloendothelial system uptake. Additionally, there have only been a few clinical investigations on XG-based formulations, and more should be undertaken to leverage XG’s exciting features. For instance, XG has been used in a clinical trial for oral delivery of budesonide (NCT02125851). In another study, the clinical efficacy of XG-based hydrogels (2%) containing lidocaine and prilocaine-encapsulated lipid nanoparticles has been studied for local application to palatine mucosa (NCT05912335).

## Figures and Tables

**Table 1:** Properties of commercial XG.

Appearance	Off-white to pale yellow, free-flowing powder
Melting point	64.43 °C
Moisture content	8–15%
Ash	7–12%
Solubility	Highly soluble in both cold and hot water, practically insoluble in organic solvents
Viscosity	13–35 cP (1 g/L solution at 25 °C)
pH	6.0 to 8.0 for a 10 g/L solution
LogP	3.926 (estimated)

**Table 2:** XG-based formulations for sustained delivery of oral therapeutics.

Polymer Matrix	Formulation Type	Formulation Composition	Drug	Key Features	Reference
XG	Matrix tablet	20, 40, and 60% *w*/*w*	Propranolol hydrochloride	Drug release was prolonged for up to 24 h. The drug release follows the Higuchi model.	[41]
XG (Mn of 2000 KDa–16,000 KDa)	Tablet	0, 25 and 40% *w*/*w*	Quetiapine fumarate	XG formed a gel layer on the tablet surface. Tablets showed extended drug release comparable to the reference product.	[42]
XG (1% aqueous solution viscosity of 1350 cps at 25 °C)	Matrix tablet	14.2%, 19%, 23.8%, 28.57%, 33.3%, and 38% *w*/*w*	Isosorbide-5-mononitrate	33.3% *w*/*w* XG containing tablets demonstrated a superior sustained release profile, with 92.12% of the drug being released over 12 h. Drug release follows the Higuchi model.	[10]
XG	Matrix tablet	3.4, 6, 12.1, 17.2, and 29.3% *w*/*w*	Pentoxifylline	The drug release rate decreased with increasing XG concentration. A greater drug release rate was evident ah pH 1.2 compared with pH 7.4.	[12]
XG-graft-poly (N-vinyl-2-pyrrolidone) (XG-g-PNVP)	Microbead	Polymer to drug ratio 10:1	Levofloxacin	Sodium alginate cross-linked XG-g-PNVP copolymer beads demonstrate high entrapment efficiency (62%) of the water-soluble drug levofloxacin. Controlled levofloxacin delivery with ~80% of the antibiotic being released in PBS (pH 7.4) media within 36 h.	[51]
Thiolated XG	Disc	Polymer to drug ratio 2:1	Tannic acid	Thiolation improved the mucoadhesive strength of XG. Modified XG exhibited a more sustained release of tannic acid than the unmodified polymer.	[52]
Thiolated XG	Matrix tablet	14.1, 17.5, and 20.8% *w*/*w*	Lornoxicam	Thiolated XG had a high swelling index. Modified XG demonstrated a sustained release of lornoxicam in a PBS (pH 6.8) release medium compared to unmodified XG.	[53]
N-trimethyl chitosan/CMXG (1:1 weight ratio)	Hydrogel	Drug to hydrogel ratio ranging from 0.44:99.56 to 3.52:96.48	Ciprofloxacin	Facilitated zero-order drug release Released ciprofloxacin has shown promising efficacy against both Gram-positive and Gram-negative bacteria.	[54]
Galactomannan/XG (0:10, 3:7, 5:5, 7:3, and 10:0 weight ratios)	Matrix tablet	10% *w*/*w*	Theophylline	Synergistic interactions between galactomannan and XG significantly delayed the theophylline release rate. In vitro drug release from matrices confirmed an anomalous transport mechanism.	[50]
XG/HPMC	Matrix tablet	Combination of XG (10–20% *w*/*w*) and HPMC (2.75–4.25% *w*/*w*)	Ranolazine	Polymer combination showed comparable drug release profiles with the commercial extended-release tablets. The use of this combination of polymers improved the biological half-life, bioavailability, and MRT of ranolazine.	[55]
XG (Mw about 2000 kDa)/chitosan (Mw 28 kD, degree of deacetylation of 89%)	Nanofiber	Electrospun solution consists of XG (0.75% *w*/*v*), chitosan (3% *w*/*v*), and curcumin (2% *w*/*v*)	Curcumin	Synthesis of stable XG/chitosan-based electrospun nanofibers in an aqueous medium. pH-dependent curcumin release with a higher release at neutral media (~50%) and lower release at pH 2.2 (~20%) after 5 days.	[56]

**Table 3:** XG-based gastroretentive drug delivery systems.

Polymer Matrix	Formulation Type	Drug	Key Features	Reference
XG, HPMC K15, and HPMC K100M	Floating matrix tablet	Stavudine	Floating tablets showed acceptable pre-compression and post-compression characteristics. Sustained release of the antiviral drug stavudine was attained with all the developed formulations.	[73]
XG	Floating tablets	Diltiazem hydrochloride	Tablets prepared with a drug/polymer ratio of 1:3 demonstrated acceptable outcomes regarding prolonged drug release and floating lag time. Formulated tablets showed satisfactory physical stability when stored at 40 °C under 75% RH for 3 months.	[74]
Carbopol 934P/XG	Mucoadhesive patch	Levetiracetam	The patch system showed excellent swelling capacity, adequate mucoadhesive properties, and unfolding behavior. It enabled the sustained release of levetiracetam for 12 h.	[75]
Eudragit/XG	Buoyant microsponge	Ranitidine hydrochloride	Developed microsponges showed suitable in vitro performance. In albino rats, ranitidine microsponges had better antiulcer activity than pure ranitidine.	[76]
XG/HPMC	Floating matrix tablet	Salbutamol sulphate	Formulation factors such as polymer type, polymer concentration, polymer ratio, and NaHCO_3_ concentration significantly affected release rate, cumulative release at one hour, and floating lag time but not floating duration. Tablets with 24.79% of XG/HPMC (1:3) and 5% NaHCO_3_ remained buoyant in the stomach fluid and steadily released the drug over 12 h.	[77]
XG/guar gum	Floating matrix tablet	Atenolol	The manufactured tablets were capable of floating over 12 h and offered a sustained drug release profile. Tablets were retained in the rabbit’s stomach for more than 6 h.	[78]
Chitosan/XG	Floating beads	Glipizide	The floating and mucoadhesive characteristics of the beads can be utilized to target drug release in the upper portion of the small intestine. The swelling of beads was reduced under acidic settings, thereby protecting drug molecules that disintegrate in acidic conditions in the stomach.	[79]
Carbopol 934/XG	Floating matrix tablet	Domperidone	Tablets prepared with the Carbopol/XG polymer combination had higher total floating time than those with XG alone. Capable of extending drug release for more than 12 h	[80]
XG/HPMC K100M CR/polyethylene oxide coagulant/carbopol 974P	Floating and gastric bioadhesive minimatrices	Amoxicillin	Sustained release amoxicillin gastroretentive mini-matrices were formulated and optimized utilizing a central composite design. The optimized formulation with the most independent variables had a buoyancy lag time of 7 min, and the drug release at one hour was 32.5%, while 95% of the drug was released in 9.39 h.	[81]

**Table 4:** XG-based formulations for colon-specific delivery.

Polymer Matrix	Formulation Type	Drug	Key Features	Reference
XG	Microspheres	α-linolenic acid (ALA)	ALA was effectively encapsulated into the microsphere. Microsphere-encapsulated ALA more efficiently inhibited colorectal cancer cell growth than the free ALA at the same concentration.	[98]
Cross-linked XG–starch	Hydrogel	Aspirin, paracetamol	Drug release was substantially greater at pH 7.4 compared to the acidic and neutral medium. The synthesized hydrogel was nontoxic, hemocompatible, and suitable for colon-specific drug delivery.	[99]
XG/guar gum	Compression-coated tablets	Metronidazole	The drug content of the compression-coated tablets was determined to be 97.5%. After 24 h of dissolution in the human fecal medium, the cumulative drug release from the 250 mg compression-coated tablets was around 84.8 ± 1.22%.	[100]
Chitosan/XG	Microparticle-based tablets	Quercetin	The microparticles were successfully loaded with quercetin and were smooth, spherical, and approximately 5 μm. The drug release followed non-Fickian diffusion.	[101]
Konjac glucomannan/XG	Tablets	Diltiazem	The drug release from the tablets was zero-order, but it was augmented in the presence of *A. niger* β-mannanase at levels comparable to those found in the colons of humans. Nonetheless, notable differences between the Japanese and American KGM varieties concerning the extent of acetylation and particle size resulted in significant variations in swelling rate and drug release between formulations made with one and the other KGM.	[102]
Konjac glucomannan/XG	Matrix tablets	Cimetidine	The drug release data demonstrated that the synergistic interaction between KGM and XG in the gel phase effectively retarded drug diffusion. The incorporation of β-mannanase accelerated the drug release rate from the KGM-containing matrices but did not affect tablets prepared by XG alone.	[16]
XG/guar gum and XG/guar gum/chitosan	Compression-coated tablets	Secnidazole	Tablets containing a single polymer in the coating layer were inappropriate for releasing secnidazole into the colon. Compression coating with chitosan (50 mg) and XG or guar gum in equal proportions (75 mg each) was more likely to enable colon-targeted delivery of secnidazole.	[103]
Konjac gum, XG, and sodium alginate	Hydrogel	Hydrocortisone sodium succinate	The hydrogel formulation demonstrated sustained release properties with low drug release at pH 1.2 (23.40% in 2 h) and pH 6.8 (25.88% in 4 h) and higher release at pH 7.4 (70.20% in 4 h). Hydrogel was found to be non-toxic, decreased spleen and thymus index, and had a clear therapeutic effect on ulcerative colitis.	[104]
XG/pluronic F-127	Hydrogel	Atomoxetine HCl	Higher drug release was seen at pH 7.4 compared to pH 1.2 and 4.6, respectively. Drug release from the hydrogels followed the first-order release kinetics.	[105]
CMXG/sodium alginate	Compression-coated tablets	Flurbiprofen	Compression-coated tablets facilitated chronotherapeutic properties with a lag time of 5.99 h and T90% of 11.48 h. In vivo pharmacokinetic studies in healthy male New Zealand rabbits with chronotherapeutic drug delivery with a lag time of 6 h	[106]
